# Platelet-Derived Growth Factor in the Ovarian Follicle Attracts the Stromal Cells of the Fallopian Tube Fimbriae

**DOI:** 10.1371/journal.pone.0158266

**Published:** 2016-07-05

**Authors:** Ching-Hua Yeh, Pao-Chu Chen, Chiu-Hua Chen, Che-Fang Hsu, Rui-Len Huang, Dah-Ching Ding, Tang-Yuan Chu

**Affiliations:** 1 Cervical Cancer Prevention Center, Tzu Chi General Hospital, Hualien, Hualien, 970, Taiwan, ROC; 2 Department of Obstetrics and Gynecology,Tzu Chi General Hospital, Hualien, Hualien, 970, Taiwan, ROC; 3 Department of Obstetrics and Gynecology, Shuang-Ho Hospital, Taipei Medical University, New Taipei City, Taiwan, ROC; 4 Institute of Medical Sciences, Tzu Chi University, Hualien, 970, Taiwan, ROC; University of Quebec at Trois-Rivieres, CANADA

## Abstract

During human ovulation, the fallopian tube fimbriae must move to the ovulation site to catch the oocyte. As the tissue-of-origin of the majority of ovarian high-grade serous carcinoma (HGSC), the fallopian tube fimbriae carrying a precursor cancer lesion may also approach the ovulatory site for metastasis. We hypothesize that platelet-derived growth factor (PDGF) in mature follicle fluid (FF) attracts the migration of PDGFR-expressing fimbriae toward the ovulating follicle. We observed that more PDGFR-β was expressed in the distal part than in the proximal parts of the fallopian tube, particularly in stromal cells in the lamina propria. The stromal cells, but not the epithelial cells, from normal fimbriae and fallopian tube HGSC were highly chemotactic to mature FF. The chemotactic activities were positively correlated with PDGF-BB and estradiol levels in FF and were abolished by a blocking antibody of PDGFR-β and by tyrosine kinase inhibitor imatinib. When PDGF-BB/AB was depleted from the FF, more than 80% of chemotaxis activities were diminished. This study suggests an ovarian follicle-directed and PDGF-dependent attraction of fallopian tube fimbriae before ovulation. The same mechanism may also be crucial for the ovarian homing of HGSC, which largely originates in the fimbriae.

## Introduction

Before fallopian tube fimbriae can pick up the cumulus oophorus, they must migrate to the location of ovulation in advance. The mechanism of this movement is illusive. After the LH surge and before ovulation, cellular and extracellular matrix layers at the follicular apex undergo thinning, and the basement membrane is degraded through proteolysis. These signs of human ovulation have been visualized through laparoscopy and described as a “follicular stigma” [1–3]. At the time of ovulation, the fimbriae exhibit an erectile extension, which is arranged over the ovulating follicle, and the vessels are engorged [4].

The fallopian tube fimbriae exhibit a mucosal lining comprising a single columnar epithelial layer and the lamina propria. In contrast to the proximal parts of the tube that possess multiple smooth muscle layers, the smooth muscle is thin and sparse in the fimbriae. We hypothesized that a chemoattractant released from the ovulating follicle is responsible for the approaching of the fimbriae to the ovulation site for oocyte pickup.

Increasing evidence indicates that the fallopian tube fimbriae, rather than the ovarian surface epithelium,are the origin of the majority of ovarian high-grade serous carcinoma (HGSC), which constitutes the major and most destructive type of ovarian cancer [5,6]. Gene expression arrays have indicated that ovarian HGSC exhibits a greater resemblance to the epithelium of the fimbriae than to that of the ovarian surface [7]. The long-sought-for precursor of ovarian HGSC appears to develop from an occult carcinoma in the fimbriae and is designated as serous tubal intraepithelial carcinoma (STIC) [8,9].

Through an unknown mechanism, STIC eventually translocates to the adjacent ovary and develops into ovarian HGSC. We suppose that a chemotaxis of the fimbria stroma toward the ovulating follicle may facilitate the movement of fallopian tube fimbriae to the ovulation site, aiding oocyte pickup as well as the ovarian homing of STIC. This study primarily focused on determining whether normal- or cancer-associated stromal cells of the fallopian tube undergo ovulation-driven chemotaxis andon identifying the mechanism of this chemotaxis.

## Materials and Methods

### Clinical Specimens

The procurement of tissue specimens and body fluids was approved by the Institutional Review Board of Tzu Chi General Hospital, Hualien, Taiwan (TCGH-IRB102-146). Signed informed consent was provided by each donor. Three samples of normal-associated fibroblasts (NAFs) were derived from the normal fimbrial tissue of patients undergoing salpingectomy along with a main operation for mature ovarian teratoma (FTSC15) or uterine myoma (FTSC22, FTE27). The primary cancer tissues of 3 cases of HGSC of the fallopian tube with various degrees of differentiation, invasion, and peritoneal spreading (Table 1) were also procured and cultured to obtain cancer-associated fibroblasts (CAFs). Table 1 presents a summary of the clinical data of these donors. Among the CAFs, FTCa1 was derived from a locally confined, serous carcinoma in the fimbriae with microscopic seedings on the omentum; FTCa9 was derived from an anaplastic carcinoma with lymphatic metastasis and peritoneal seeding; and FTCa12 was derived from a moderately differentiated carcinoma with lymphatic and peritoneal spreading. A total of17 follicular fluid (FF) aspirates were procured from the remaining materials of the in vitro fertilization program at Tzu Chi General Hospital, according to a protocol described earlier [10]. Normal fallopian tube tissue was obtained from cases who underwent salpingectomy during operation for benign tumors of the uterus [1].

**Table 1:** Clinical characteristics of patients with normal and malignant fallopian tub fimbriae.

**Cell name**	**FTSC15**	**FTSC22**	**FTE27**
Age	47	44	50
Menstrual phase	Postmenopause	Proliferative phase	Early luteal phase
Diagnosis	Mature teratoma of ovary	Uteirne myoma	Uterine myoma
Cell culture	Fimbriae stromal cells	Fimbriae stromal cells	Fimbriae epithelial cells
**Cell name**	**FTCa1**	**FTCa9**	**FTCa12**
Age of patient	68	52	63
Histology	Serous carcinoma of left fallopian tube, with bilateral STIC	Serous carcinoma	Serous carcinoma
Differentiation	Well to poor	Anaplastic	Moderate
FIGO stage (TNM)	3A (T2aN0M0)	3C (T2cN1M0)	3C (T3cN1M0)
Stromal invasion	Superficial	Overt	Overt
Regional involvement	None, only LVSI	Right ovarian hila and infundibulopelvic ligament, uterine vascular lumen	Bilateral ovaries and uterus
Intra-abdominal spreading	Omentum (microscopic)	Negative	Omentum, peritoneum (overt)
Metastasis	Negative	Para-aortic LN	Para-aortic LN
CA125 (U/ml)	21.2	50.4	987
P53 mutation	275C>F (exon 8)	No mutation	NA
P53 stain in nucleus	53/75 cells (71%)	Negative	NA

Abbreviation: STIC: serous tubal intraepithlial carcinoma,

LVSI: lympho-vascular invasion,

LN: lymph node

NA: non-applicable

### Primary Culture of Stromal Cells of the Normal Fimbriae and Fallopian Tube Carcinoma Specimens

For primary culture of tubal stromal cells, the procured tissue was cut and treated with an erythrocyte lysis buffer (160 mM of NH_4_Cl) for 10 min. The tissue was then digested with type I collagenase (5 mg/g, Sigma-Aldrich, St. Louis, MO) and dispase (4 units/g, Gibco BRL, Gaithersburg, MD) for 1 h at 37°C. The isolated cells were cultured in DMEM low glucose/KSFM (1:1) medium (Gibco BRL, Gaithersburg, MD) with 5% fetal bovine serum (Hyclone Laboratories, Logan, UT), 50 mg/mL of sodium ascorbate, N-acetyl-L-cysteine (Sigma-Aldrich, St. Louis, MO), and antibiotics (100 U/mL each of penicillin G and streptomycin) in a CO_2_ incubator with medium change every other day. Cell spreading was typically observed 6 h after seeding and was maintained at subconfluent levels. For the NAF culture, the tissue was seeded in a 6-well dish and then transferred to a 10-cm dish. The third to fourth passages of cells were used for the experiments. For the CAF culture, the tissue was seeded in a 24-well dish and passed to a 6-well dish and to a 10-cm dish. The eighth to tenth passages of cells were used for experiments.

### Pyrosequencing Analysis of the G824T Mutation of TP53

To assess the extent of contamination of the cancer cells in the FTCa1 cell culture, we identified a G824T mutation of the *TP53* gene in the carcinoma tissue from which FTCa1 was derived. The quantity of this mutation was determined through quantitative pyrosequencing. In brief, the primers were designed using PyroMark Assay Design 2.0 Software. The sequencing regions covered 5’-TGTTTGTGCCT[G/T]TCCTGGGAGA-3’, which spans the G824T mutation site of *TP53*. The amplification primers were a forward primer (5’-TACTGGGACGGAACAGCTTTGAG-3’) and a biotinylated reverse primer (5’-TTGCGG AGATTCTCTTCCTCTGT-3’). The sequencing primer was 5’-TTTGAGGTGCGT GTTT-3’. PCR was performed using 20 ng of genomic DNA, 450-nM primers, and 20 μL of 1X PCR Master Mix under the following conditions: denaturation at 95°C for 15 min, 45 polymerization cycles (at 95°C for 30 s, 60°C for 40 s, and 72°C for 40 s), and final extension at 72°C for 5 min. The PCR products were purified and mixed with a sequencing primer (0.3 μM) and subjected to an AQ pyrosequencing assay using the PyroMark Q24 system (QIAGEN, MD).

### Immunocytochemistry and Immunohistochemistry

For immunocytochemistry and immunohistochemistry, cells were harvested and fixed on glass slides, and tissue was fixed, paraffin embedded, and sectioned at a thickness of 5 μm. Immunostaining was conducted using antibodies for vimentin and platelet-derived growth factor receptor (PDGFR)-β (both from Abcam, UK), each at a dilution of 1:200. The slides were treated with an antibody for 1 h at room temperature (RT), washed 3 times with a PBS-T buffer (10 mM sodium phosphate, 0.15 M NaCl, 0.05% Tween-20, pH 7.5) for 5 min each, and subjected to biotin-labeled secondary antibodies (1:1000 dilution) for 1 hand DAB staining for 5–10 min. For immunofluorescence analysis, the sections were treated with the aforementioned primary antibody and stained with rhodamine-conjugated secondary antibodies for PDGFR-β and fluorescein isothiocyanate-conjugated secondary antibodies (ThermoFisher Scientific, Waltham, MA) for α-SMA for approximately 1 h. After 3 washes with PBS-T, they were assessed through fluorescence microscopy.

### Flow Cytometry for Cell Surface Markers

For flow cytometry analysis, 10^6^ cells were detached using 2 mM EDTA in PBS, washed with PBS containing 2% BSA and 0.1% sodium azide (Sigma, St. Louis, MO), and incubated with the respective cell surface antibody conjugated with fluorescein isothiocyanate or phycoerythrin (ThermoFisher Scientific, Waltham, MA). Cell surface markers including CD29, CD34, CD44, CD45, and CD90 were analyzed by using the FACS Aria II Cell Sorter (BD Biosciences, San Jose, CA).

### ELISA and Western Blot Analysis

The estradiol (E2), platelet-derived growth factor (PDGF)-AA, and PDGF-BB levels in FF were measured using ELISA kits according to the protocols of the manufacturer (Abcam, UK). In brief, after the coating of ELISA wells with ligand-specific antibodies, samples and standards were added to wells in triplicate, incubated at RT for 2 h, washed 4 times, and detected using avidin-HRP-conjugated antibodies at a dilution of 1:2000 in an ELISA reader. For Western blot analysis, total protein was extracted from 1 × 10^6^ cells or the ground tissue by using a RIPA buffer (ThermoFisher Scientific, Waltham, MA), and the protein concentrations were measured through the Bradford method (Bio-Rad, CA). Samples containing 12.5 μg of total protein were resolved in SDS-PAGE, transferred to a nitrocellulose membrane, and reacted with the primary antibody for PDGFR-α, PDGFR-β (Abcam, CA), or β-actin (Sigma-Aldrich, St. Louis, MO), all at a 1:2000 dilution, and with a horseradish-peroxidase-conjugated secondary antibody (Amersham Bioscience, NJ) at a 1:2000 dilution. After being washed 3 times, the membrane was reacted with an ECL reagent (Amersham Bioscience, NJ) and exposed to X-ray film.

### Boyden Chamber Chemotaxis Assay, Blocking Antibody, and Platelet-Derived Growth Factorreceptor Fc-Sequestration

For a chemotaxis migration assay, 1300 cells were seeded on the top wells of a 6-well Boyden chamber with a membrane insert of 8-um pore size. The lower wells were filled with 30μl of tested chemoattractant (pure FF or diluted growth factors), or with serum-free culture medium as the background migration control. After 16 h, the cells on the membrane insert were fixed, stained with Giemsa, and counted. The chemotactic activity was defined as the number migrated cells minus toward the chemoattractant minus that of the background control. To assess the role of PDGFRs in the chemotaxis, imatinib (Novartis, Switzerland) or a PDGFR-β blocking antibody (Abcam, UK) was added to the top well before the assay. To deplete the PDGF ligands, FF was treated with a recombinant human PDGFR-β Fc chimera (R&D Systems, MN) at a concentration of 3–24 ng/mL for 30 min before it was added into the lower well for the chemotaxis assay.

### Statistical Analysis

GraphPad Prism software was used for statistical analyses and data plotting. Comparisons between groups were analyzed using paired or unpaired *t* tests. One-way ANOVA was performed to compare the chemotaxis:migration ratio among the groups.

## Results

### Characterization of Normal and Cancer-Associated Fallopian Tube Fimbrial Stromal Cells

**Fig 1:**
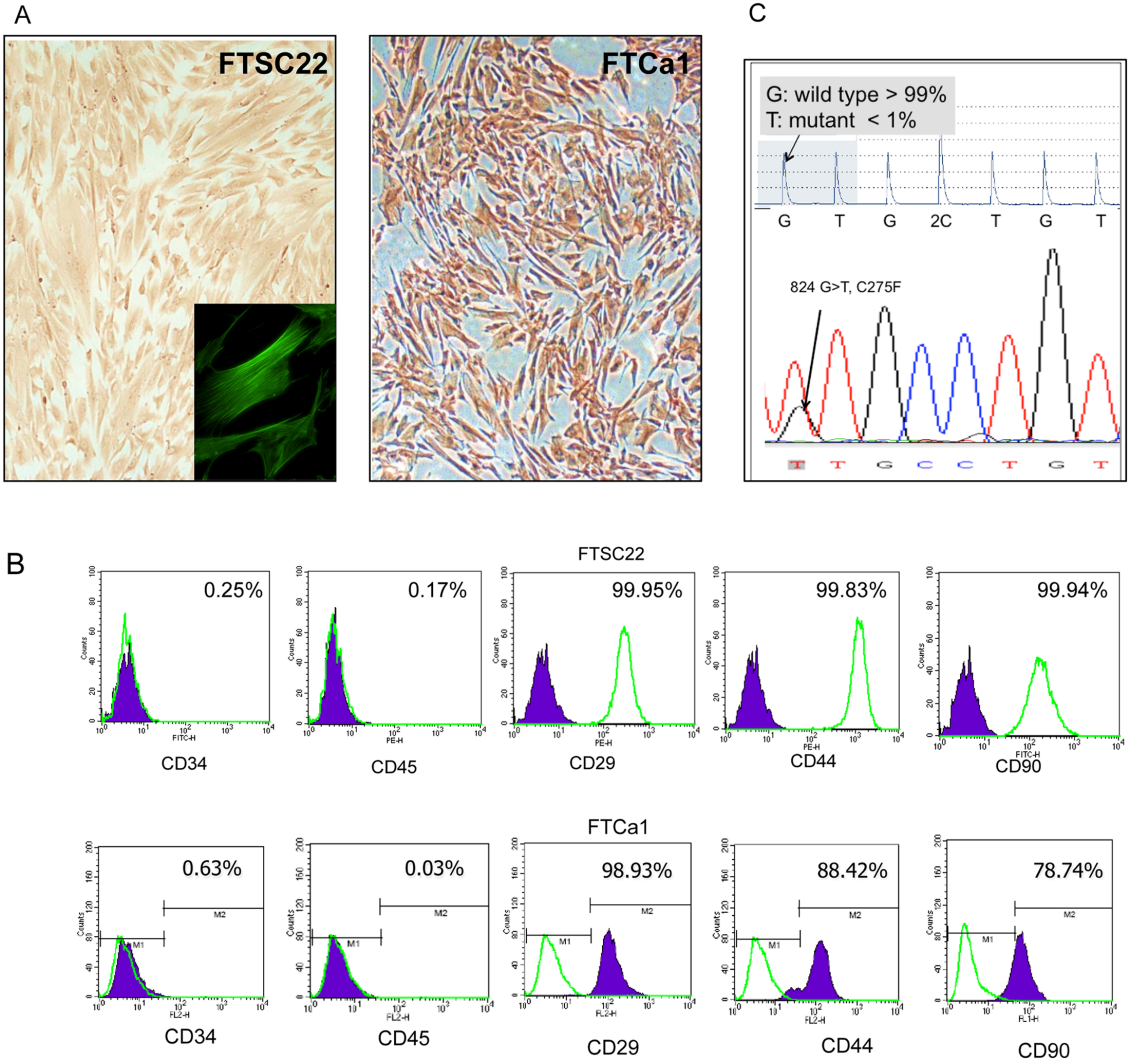
Characteristics of the normal- and cancer-associated stromal cells of the fallopian tube. (A) IHC and immunofluorescence (inserted figure) stains of vimentin in cultured NAFs (represented by FTSC22) at passage 4 and the CAFs (represented by FTCa1) at passage 8 were showed. (B) Flowcytometry analysis of expression of cell surface markers in representative NAFs and CAFs. (C) The *TP53*sequences including the 824 G>T mutation in the carcinoma tissue where FTCa1 was derived (lower panel, regular sequencing) and in FTCa1 cells (upper panel, pyrosequencing) were showed.

The NAFs from the normal human fallopian tube fimbriae and the CAFs from the fallopian tube carcinoma were universally positive for vimentin (Fig 1A). These stromal cells were both negative for CD34 and CD45 and positive for CD29, CD44, and CD90, indicating the characteristics of mesenchymal cells (Fig 1B). To rule out the presence of cancer cells in the CAF culture, we subjected the FTCa1 CAF and original carcinoma tissue, which had been determined to possess a*TP53* 824 G>T mutation, to pyrosequencing to quantitate the mutation allele. The results indicated that 99% of the FTCa1 cells were negative for this mutation (Fig 1C).

### PDGF-Dependent Chemotaxis of NAFs toward Mature FF

**Fig 2:**
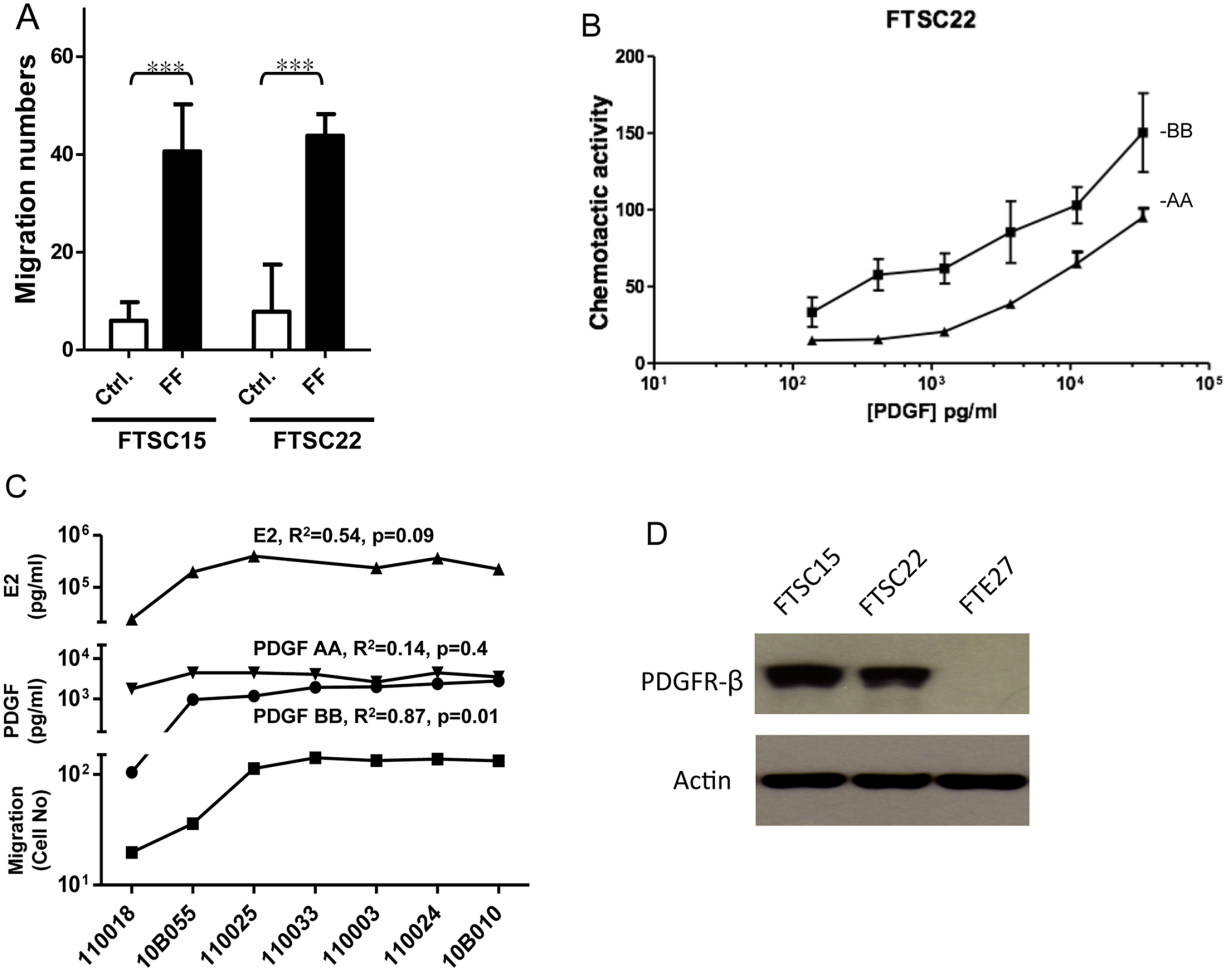
Stromal cells of normal fimbriae undergo chemotaxis to mature follicular fluid as well as PDGF. (A) Boyden chamber migration assay of NAFs derived from 2 normal fimbriae (FTSC15 and FTSC22) toward the control medium (DMEM with 5% fetal bovine serum, Ctrl.) and a pool of 4 FFs (specimen ID:110018, 10B055, 110025 and 110033). ***: *p*< 0.001. (B) Chemotaxis activities of FTSC22 cells toward PDBF-BB and PDGF-AA of different concentrations. The chemotaxis cell number was calculated by subtracting the migration cell number of control medium from that of chemoattractant. (C) Levels of estradiol (E2), PDGF-AA, PDGF-BB, as well as migration cell number of FTSC22 toward seven different FFs (specimen ID indicated at X-axis). Each datum was obtained from an experiment that was repeated 6 times. The error bars were too small to be displayed in the figure. (D) Western blot analysis of PDGFR-β expression in the 2 NAFs (FTSC15 and FTSC22) and a primarily cultured-fimbrial epithelial cells (FTE27).

The NAFs derived from the 2 normal fimbriae (FTSC15 and FTSC22) exhibited similar migration activity in the control medium and 5–7 times more activity toward a pool of FFs (both *p*< 0.0001; Fig 2A). Among the various candidate chemoattractants reported previously in mature human FF [3, 11], we evaluated the chemotactic activity of PDGF-BB, PDGF-AA, PDGF-AB, PDGF-DD, kit ligand (SCF), and IGF. Only PDGF-BB and PDGF-AA exhibited dose-dependent chemotactic activity, and PDGF-BB activity was 1.6- to 3.7-fold higher than PDGF-AA activity (Fig 2B and data not shown). To investigate the relationship between chemotaxis activity and the PDGF and estradiol levels in FFs, 7 different FFs were each analyzed. As showed Fig 2C, the FF-attracted migration cell number exhibited a positive correlation with the PDGF-BB (R^2^ = 0.87, *p* = 0.01) but not the PDGF-AA level (R^2^ = 0.14, *p* = 0.40) in the FF (Fig 2C). In addition, increased expression of PDGFR-β protein, which binds PDGF-BB and PDGF-AB, was detected in the two NAFs; by contrast, this receptor was not expressed in the primary fimbria epithelial cells (Fig 2D).

### Increased PDGFR-β Expression in the Fallopian Tube Fimbria Stroma

**Fig 3:**
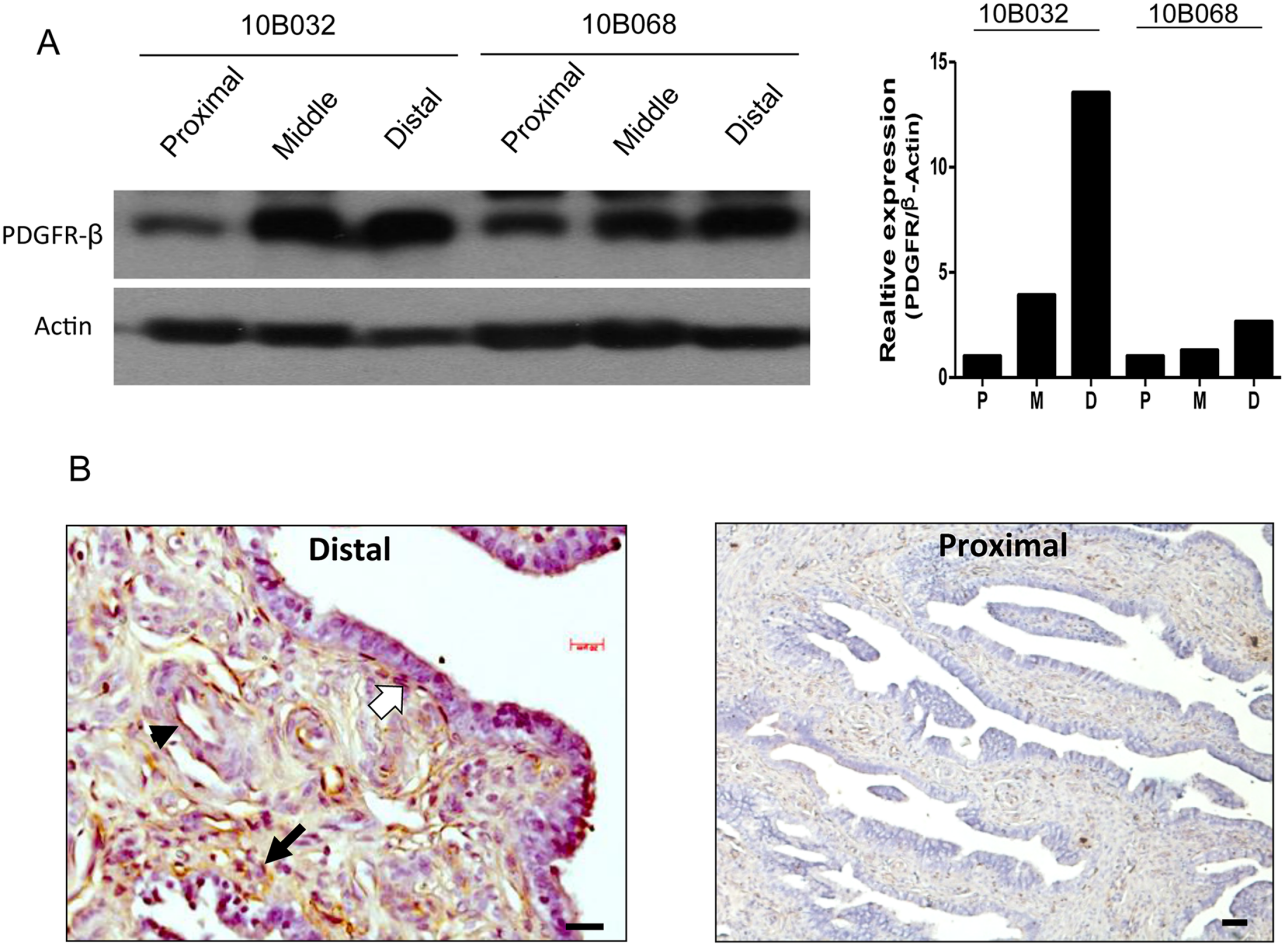
Increased expression of PDGF-β in the fimbriae of the fallopian tube. (A) Western blot analysis of PDGFR-β in various parts of a single fallopian tube from 2 healthy women. Distal: the fimbriae part, Middle: the ampula part, Proximal: the isthmus part. (B) Immunohistochemistry of PDGFR-β expression in different locations of human fimbria tissue: subepithelial (opened arrow), perivascular (arrow head) and lamina propria (black arrow). The scale bar was 50 μM.

We further investigated the localization of PDGFR-β protein in various parts of the fallopian tube. As displayed in Fig 3A, PDGFR-β protein was more expressed in the distal (fimbriae) than in the middle (ampula) and proximal (isthmus) parts of the same fallopian tube (Fig 3A). Throughimmunohistochemical examination of the fallopian tube, we determined that the expression of PDGFR-β was localized to the subepithelial region of the lamina propria (Fig 3B) as well as to the perivascular pericytes (Fig 3B).

### Cancer-Associated Fibroblasts of High-Grade Serous Carcinoma of the Fallopian Tube Undergo Chemotaxis to Follicular Fluid through PDGF/PDGFR Signaling

**Fig 4:**
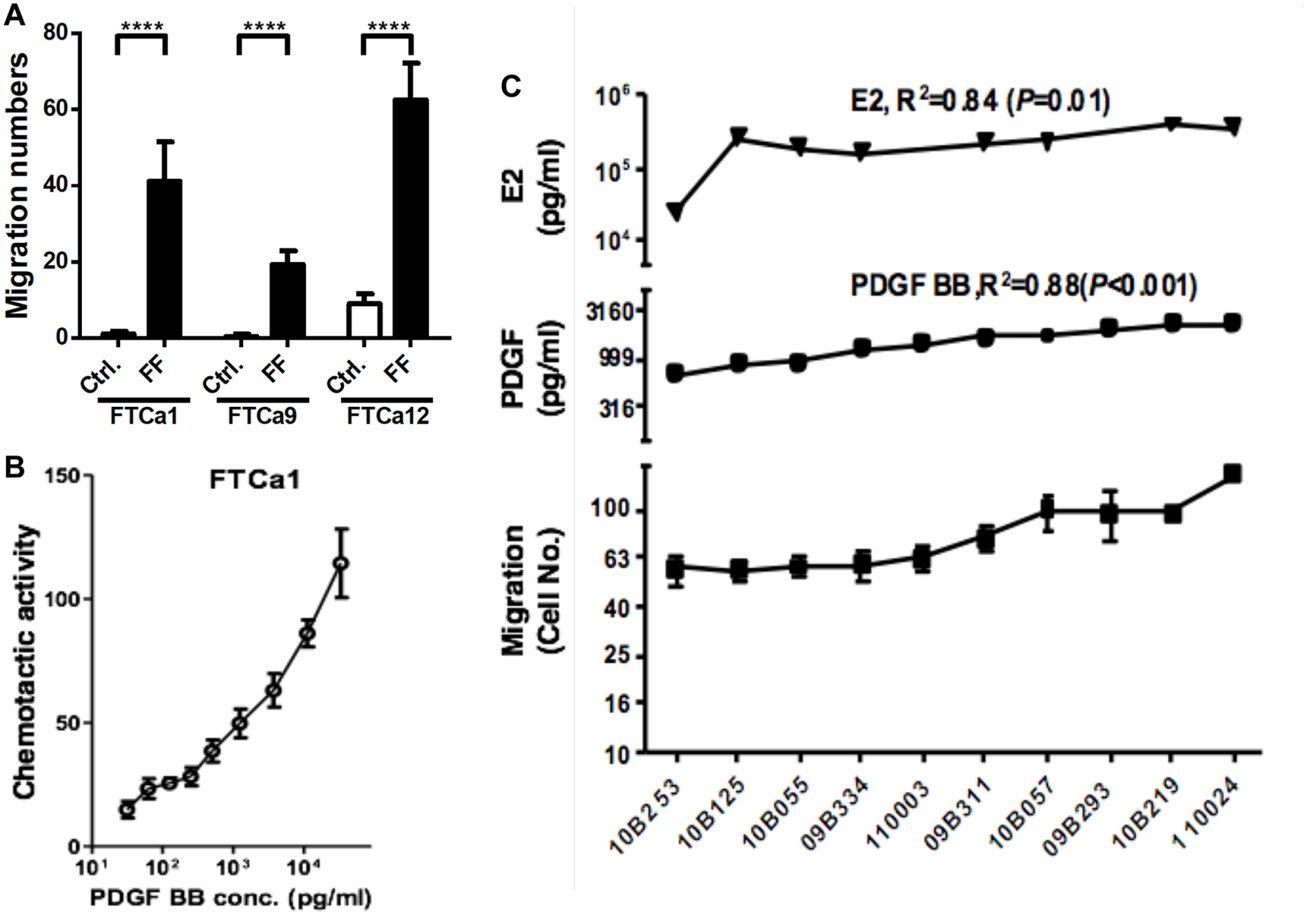
Cancer-associated fibroblasts from primary fallopian tube carcinoma undergo chemotaxis to follicular fluid. (A) Boyden chamber migration assay of CAFs toward control medium and a pool of 4FFs (specimen ID: 10B253, 10B125, 10B005 and 09B334). (B) Chemotactic activities of FTCa1 cells toward PDGF-BB of different concentrations. (C) Levels of estradiol (E2), PDGF-BB, as well as migration cell number of FTCa1cells toward ten different FFs (specimen ID indicated at X-axis). Each datum was obtained from an experiment that was repeated 6 times. ****p< 0.0001.

Because normal fimbrial stromal cells undergo chemotaxis to mature follicles in response to PDGF, we examined whether the CAFs of fallopian tube carcinoma also undergo the same chemotaxis. Owing to the extremely limited source and the microscopic size of the STIC lesions that prohibited primary culture, the CAFs of the primary HGSC tumor of the fallopian tube were used as surrogates. We enriched CAFs from 3 specimens of primary serous carcinoma of the fallopian tube with varying clinical characteristics (Table 1). All 3 CAFs exhibited strong chemotaxis toward a pooled FFs, compared witha control medium (Fig 4A). Among them, the carcinoma from which the FTCa1 cells were derived most resembled the clinical features of STIC and was further investigated. As illustrated in Fig 4B, similar to the NAFs, the FTCa1 cells exhibited dose-dependent chemotaxis toward PDGF-BB (Fig 4B). In addition, the FF-directed migration of the FTCa1 cells exhibited a positive correlation with PDGF-BB (R^2^ = 0.88, *p* < 0.001) and E2 (R^2^ = 0.84, *p* = 0.01) levels in the FF (Fig 4C).

### PDGF/PDGFR Signaling Is Responsible for the Chemotaxis of the Normal-Associated and Cancer-Associated Fibroblasts of the Fallopian Tube

**Fig 5:**
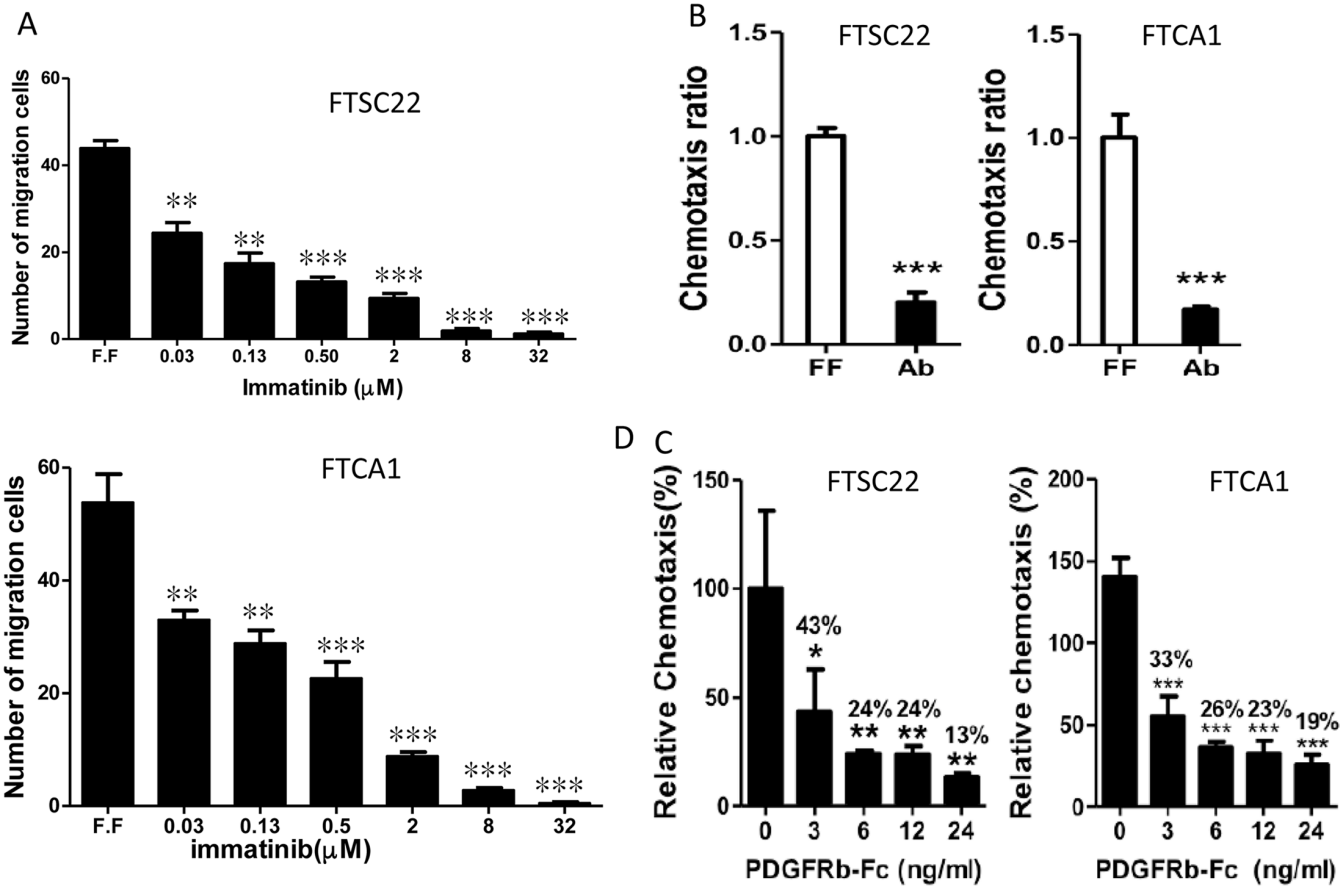
Chemotaxis of cancer-associated fibroblasts from fallopian tube carcinoma to follicular fluid depends on platelet-derived growth factor BB signaling. (A) The chemotactic activity of the FTSC22 (upper panel) and FTCa1 (lower panel) cells toward a pooled FFs (specimen ID: 10B253, 10B125, 10B005 and 09B334) was inhibited by imatinib in a dose-dependent manner. (B) Diminish of these chemotactic activities by pretreating the cells with a PDGFR-β-specific blocking antibody (Ab). (C) Chemotactic activity of the FTSC22 and FTCa1 cells after the PDGF-BB and PDGF-AB in the pooled FF were depleted using a human PDGFR-β-Fc chimera. *: *p*< 0.05, **: *p* < 0.01, ***: *p* < 0.001, according to Student's *t* test.

We further investigated the role of PDGFR signaling in the chemotaxis of fimbrial stromal cells. As shown in Fig 5A, the receptor tyrosine kinase inhibitor imatinib dose-dependently diminished the chemotaxis of both FTSC22 and FTCa1 cells (both *p* < 0.001). Moreover, treatment with a PDGFR-β-specific blocking antibody diminished the chemotactic activity in both cell types (*p* < 0.001; Fig 5B). The extent to which PDGF in FF contributes to chemotaxis was determined by depleting PDGF-BB and PDGF-AB from the FF by using the PDGFR-β-Fc chimera. After this treatment, the chemotaxis of the FTSC22 and FTCa1 cells by FF was diminished by 87% (*p* < 0.01) and 81% (*p* < 0.001), respectively (Fig 5C).

## Discussion

The study revealed a PDGFR-directed chemotaxis of stromal cells of the fallopian tube fimbriae toward mature FF. The chemotactic activity depended on the PDGF level in FF and was diminished by a blocking antibody and a tyrosine kinase inhibitor of PDGFR. This study also identified PDGF-BB as the main chemoattractant. PDGF-BB exhibited higher chemotactic activity than did PDGF-AA, and in contrast to that of PDGF-BB, the PDGF-AA levels in the FF exhibited no correlation with the chemotactic activity. When PDGF-BB/AB were depleted from the FF, the chemotactic activity of the normal and cancerous stromal cells of the fallopian tube were diminished by 87% and 81%, respectively. The results suggest that PDGF-BB or -AB is the main chemoattractant in FF. Moreover, this chemotactic activity exhibited a positive relationship with E2 levels in the FF, indicating that the chemotaxis is associated with the maturation of the ovarian follicle.

A major function of the fallopian tube fimbriae is to capture the oocyte. Before accomplishing this, they must migrate to the ovulation site through an unknown mechanism. Gordits et al. observed this migration process through endoscopy and described it as follows: “The fimbriae were distended and embracing the caudal pole of the ovary. The fimbrial vessels were engorged, and the edges of the erect fimbriae were in close contact with the ovary” [1]. This and other morphological observations [2, 3] suggest a chemotactic direction of migration of fallopian tube fimbriae to the ovulation site. We hypothesized that PDGF-directed chemotaxis may be responsible for the movement of the fallopian tube fimbriae toward the ovulating follicle.

As observed in this and other studies [12, 13], the preovulatory follicle contains an extraordinarily high PDGF level. The average level is 10 times and 25 times higher than those in the serum and peritoneal fluid, respectively [12, 13]. During ovarian folliculogenesis, granulosa cells begin secreting PDGF into follicles [11, 12, 14, 15]. After the LH surge and before ovulation, cellular and extracellular matrix layers at the follicular apex become congested and undergo thinning with drastic inflammation and angiogenesis [3, 12, 13, 16]. As one of the major inflammatory growth factors, PDGF is likely to be secreted from this inflammatory lesion into the peritoneum cavity and acts on the fimbriae.

It is also known that PDGF can open the tight junction between epithelial cells and increase extracellular permeability [13, 17]. When cells were treated with PDGF, tight junction proteins moved from the cell border to the cytoplasm, and the pericellular permeability increased in a concentration-dependent manner [13, 17]. Thus, PDGF may open the cell–cell junction of the fimbrial epithelium and act on the stromal cells in the lamina propria of the fimbriae. The study revealed PDGF-BB as the main chemoattractant in mature FF to attract normal and cancerous stromal cells in the fallopian tube fimbriae. We speculate that coordinated tissue migration could be driven by stromal cells. PDGFR signaling is essential for the migration and differentiation of various cell populations in development, such as the spreading of neural crest mesenchymal cells toward the branchial pouches, of oligodendrocyte precursors in the spinal cord, and of pericytes along newly formed angiogenic sprouts [12, 18–20]. The paracrine chemotaxis of fimbrial stromal cells induced by PDGF in FF is reminiscent of the developmental process in *Drosophila*, wherein the PDGF-equivalent PVF1 that is expressed in oocytes guides the migration of border cells that express the receptor and leads the egg chamber to move in aggregate [14, 21]. Whether the chemotaxis of the stromal cells in the fallopian tube fimbriae could drive migration toward the ovulation site is still unknown and awaits further investigations *in vivo* or in the organoid [22].

Increasing evidence indicates that ovarian HGSC originates in the fallopian tube. Fimbrial STIC has been identified as the precursor lesion of ovarian HGSC [23–25]. The most likely location and timing of implantation of STIC into the ovary is a wound of ovulation. Several large-scale epidemiological studies have reported that inhibition of ovulation reduces the incidence of ovarian cancer [26]. In this study, we illustrated that mature FF attracted both NAFs and CAFs of the fimbriae. If the theory of FF PDGF-directed fimbriae approaching the ovary is true, then through the same mechanism, fallopian tube fimbriae carrying STIC can migrate to the ovulation site, thereby enabling physical contact with the ovulation wound and seeding.

Whether the CAFs of the primary HGSC of the fallopian tube are equivalent to those associated with the STIC lesion is uncertain. Because of the extremely small size of STIC and lack of a cell model, stromal cells associated with STIC are not readily available. We suppose that the characteristics of the stroma of STIC should be between those of the stroma of normal fimbriae and those of the stroma of tubal HGSC. Because both normal and carcinomatous stromal cells of the fimbriae are chemotactic to the FF, the stromal cells of the intermediate lesion, i.e. STIC,is very likely to be chemotactic as well.

In summary, this study determined that PDGF-BB in a mature ovarian follicle is a major chemoattractant of the stromal cells of normal and cancerous fimbriae. The results disclosed a possible mechanism of fimbriae-approaching for oocyte pickup and may provide a new target of prevention for ovarian HGSC.
